# Early Steroid Withdrawal in Kidney Transplant Recipients: PRO

**DOI:** 10.34067/KID.0000000000000326

**Published:** 2025-02-27

**Authors:** Sarthak Virmani, William S. Asch

**Affiliations:** 1 University of Virginia School of Medicine, Charlottesville, Virginia; 2 Yale University School of Medicine, New Haven, Connecticut

**Keywords:** immunosuppression, kidney transplantation, renal transplantation, transplant outcomes

The use of corticosteroids to prevent organ rejection was a pioneering advancement of the 1960s and has remained a deeply rooted approach in kidney transplantation. Yet, patients routinely report their concerns for corticosteroid (hereafter, referred to simply as steroid)-induced side effects and their desire to discontinue use, even preferentially over other arguably more pernicious immunosuppressive (IS) medications. When surveyed and given a risk-free choice, about 65% of kidney transplant recipients (KTR) reported they would discontinue steroid use.^1^

Previous discussions weigh the projected long-term benefits of steroid elimination against the risk of developing allograft rejection attributed to the withdrawal of steroids.^2^ In the immunologically high-risk candidates, it is clear that short-term outcomes after kidney transplantation have significantly improved with the combined use of steroids and modern maintenance IS. But despite improvements in rates of acute rejection (AR) and 1-year post transplant allograft and patient survival, the long-term outcomes have either remained unaffected or show only modest improvement, especially in low or standard immunological risk recipients.^3^ Multiple studies, using different strategies to either eliminate or reduce the KTR's cumulative exposure to steroids, with varying combinations of other IS, have been undertaken to highlight these trends. In this debate, we continue to make the case that steroids can be safely discontinued early after transplantation for appropriate patients without significant detrimental effect on long-term outcomes.

While studies have confirmed that use of steroids reduce the risk of AR during the early post-transplant period resulting in a decline in early graft loss, it is less clear whether continued steroid use is necessary to prevent rejection months or years after transplant. It is already established that early rejections, when swiftly managed, do not necessarily affect long-term allograft outcomes.^4^ Indeed, most of the AR events that occurred did so early after steroid withdrawal, were of low Banff grade, and were easily treated with steroids. There is, in fact, growing evidence that early steroid withdrawal in the appropriate population does not result in an increased risk of long-term allograft loss.^5^

Most studies investigating the continued necessity of steroids withdraw them at a specified time point. Conventionally, steroid avoidance is defined as use for seven days or less, while protocols investigating steroid withdrawal do so in the 3–6 months after transplantation. Note that in clinical practice, late steroid withdrawal is often a consideration as well. For example, in a recipient with avascular necrosis of the hip who is not a candidate for an operative repair. Few studies have investigated late steroid withdrawals. Unfortunately, the long-term consequences of maintenance steroids are all cumulative and therefore only become evident during longer-term follow-up.

Death with a functioning allograft has increased over time, presently seen in approximately 40% of KTR. While long-term studies have been unsuccessful to support the continuing need for steroids, their ongoing use certainly has a detrimental and accruing effect on overall recipient health, especially the cardiovascular and metabolic parameters (Table 1).

**Table 1:** Detrimental effects of long-term corticosteroid use

Impaired glycemic metabolism
Weight gain
Hypertension
Hyperlipidemia
Increased appetite
Osteopenia/Osteoporosis
Osteonecrosis
Cataract development
Skin fragility
Impaired wound healing
Gastric ulceration
Impaired growth (in children)
Cushingoid appearance
Increased fat deposition
Decreased muscle production
Irregular menstrual periods
Increased risk for infections
Fluctuations in mood disorders

It is well established that steroid use results in an increased risk of major adverse cardiovascular events.^6^ Cardiovascular and cerebrovascular events collectively are the cause of 22% of KTR who die with a functioning allograft. Indeed, this rate of cardiovascular death is an alarming ten-fold that for the general population.^7^

One may argue that the relative contribution of the low-dose steroid is eclipsed by that of calcineurin inhibitors. But it is also recognized that CNI (tacrolimus, in particular) in interaction with other recipient variables (such as age and body mass index) are a risk factor for post-transplant diabetes mellitus development. Recent studies have shown feasibility of simultaneous CNI avoidance and steroid withdrawal for an improved metabolic profile without excessive AR in the first 12 months after transplant.^8^

Nonetheless, steroid avoidance, early elimination, and late steroid withdrawal all have the potential to reduce the long-term risk of cardiovascular events and death with a functioning allograft, thereby extending the lifetime of the kidney transplant recipient. Therefore, it may be reasonable to accept a higher risk of early AR (which in fact may help label those recipients who have a proven need for continued steroid use) to identify the larger group of KTR who would see the benefit of a reduced cardiovascular risk by not receiving steroids long term. This magnitude of increased risk has been reported to be low in selected low immunologic risk (panel reactive antibodies ≤ 25%) patients, but it should be noted that borderline findings were not recorded as rejection in all studies investigating steroid avoidance.^9^

Were we to broadly adopt the strategy of universally attempting steroid avoidance or early withdrawal, or perhaps even late withdrawal, we need to more clearly consider the fraction of recipients who would experience a negative consequence. We must also consider how an AR event early after transplant that receives proper management affects the long-term function of these allografts. Another consideration of how much additional harm comes from a one-time high-dose (1500–2000 mg) bolus steroid use to treat AR and weigh this in comparison to the adverse effects of lifelong low-dose steroid use.

Multiple studies and two Cochrane debased reviews have investigated the effect of steroid avoidance on post-transplant outcomes (rejection and post-transplant diabetes mellitus, in particular). Using meta-analysis compared with steroid maintenance, steroid withdrawal was associated with a significantly increased 1-year relative risk of AR (RR=1.77). However, it should be noted that 1-year mortality (RR=0.68) and new-onset diabetes after transplantation (RR=0.77) were both reduced.^10^

The elderly are more susceptible to steroid-related complications compared with younger KTR, particularly to fractures, myopathy, and age-related immune dysfunction resulting in infection and malignancy.^11^ However, lower-intensity immunosuppression regimens, including steroid avoidance, are known to have equivalent allograft outcomes in elderly recipients. The effect of low-dose steroids on these negative outcomes should not be discounted, as is often the case when considered relative to higher doses. Notably, there are recommendations to prevent, reduce, or retard the development of premature or exaggerated osteoporosis for patients taking prednisone ≥7.5 mg/d, but no such recommendations have been established for prednisone 5 mg/d, the dose most commonly used as part of maintenance immunosuppression regimens. But experimental data confirm the negative effect of low-dose prednisone on markers of bone formation, which were lower compared with patients receiving placebo.^12^

We acknowledge the increased risk of rejection when steroid avoidance and steroid withdrawal protocols are used in high-risk KTR. And while 3-year allograft survival seems equivalent, we also recognize the concern for an increased rate of DSA development and the role this may have on very long-term allograft survival. Despite these valid concerns, these risks seem to be worth accepting for a carefully selected subgroup of recipients. An increased risk of early, low-grade, steroid-responsive rejection should not be an absolute contraindication to considering an early steroid avoidance, withdrawal, or minimization protocol. Moreover, given that about 12% of current US transplant waitlist comprises retransplant candidates, the long-term use of steroids should be justified by the cumulative cardiovascular and metabolic risks which unfortunately only become apparent after several years of use.

## References

[1] Prasad GV Nash MM McFarlane PA Zaltzman JS. Renal transplant recipient attitudes toward steroid use and steroid withdrawal. Clin Transplant. 2003;17(2):135–139. doi:10.1034/j.1399-0012.2003.00034.x 12709080

[2] Bae S Garonzik Wang JM Massie AB, . Early steroid withdrawal in deceased-donor kidney transplant recipients with delayed graft function. J Am Soc Nephrol. 2020;31(1):175–185. doi:10.1681/ASN.2019040416 31852720 PMC6934998

[3] Woodle ES First MR Pirsch J Shihab F Gaber AO Van Veldhuisen P. a prospective, randomized, double-blind, placebo controlled multicenter trial comparing early (7 day) corticosteroid cessation versus long-term, low-dose corticosteroid therapy. Ann Surg. 2008;248(4):564–577. doi:10.1097/SLA.0b013e318187d1da 18936569

[4] Klintmalm GB Vincenti F Kirk A. Steroid-responsive acute rejection should not be the end point for immunosuppressive trials. Am J Transplant. 2016;16(11):3077–3078. doi:10.1111/ajt.13889 27232598

[5] Woodle ES Gill JS Clark S Stewart D Alloway R First R. Early corticosteroid cessation vs long-term corticosteroid therapy in kidney transplant recipients: long-term outcomes of a randomized clinical trial. JAMA Surg. 2021;156(4):307–314. doi:10.1001/jamasurg.2020.6929 33533901 PMC7859872

[6] Pujades-Rodriguez M Morgan AW Cubbon RM Wu J. Dose-dependent oral glucocorticoid cardiovascular risks in people with immune-mediated inflammatory diseases: a population-based cohort study. PLoS Med. 2020;17(12):e1003432. doi:10.1371/journal.pmed.1003432 33270649 PMC7714202

[7] Neale J Smith AC. Cardiovascular risk factors following renal transplant. World J Transplant. 2015;5(4):183–195. doi:10.5500/wjt.v5.i4.183 26722646 PMC4689929

[8] Woodle ES Kaufman DB Shields AR, . Belatacept-based immunosuppression with simultaneous calcineurin inhibitor avoidance and early corticosteroid withdrawal: a prospective, randomized multicenter trial. Am J Transplant. 2020;20(4):1039–1055. doi:10.1111/ajt.15688 31680394

[9] Ekberg J Baid-Agrawal S Jespersen B, . A randomized controlled trial on safety of steroid avoidance in immunologically low risk kidney transplant recipients. Kidney Int Rep. 2022;7(2):259–269. doi:10.1016/j.ekir.2021.11.028 35155865 PMC8821032

[10] Haller MC Royuela A Nagler EV Pascual J Webster AC. Steroid avoidance or withdrawal for kidney transplant recipients. Cochrane Database Syst Rev. 2016;2016(8):CD005632. doi:10.1002/14651858.CD005632.pub3 27546100 PMC8520739

[11] Lentine KL Cheungpasitporn W Xiao H, . Immunosuppression regimen use and outcomes in older and younger adult kidney transplant recipients: a national registry analysis. Transplantation. 2021;105(8):1840–1849. doi:10.1097/TP.0000000000003547 33214534 PMC10576532

[12] Ton FN Gunawardene SC Lee H Neer RM. Effects of low-dose prednisone on bone metabolism. J Bone Miner Res. 2005;20(3):464–470. doi:10.1359/JBMR.041125 15746991

